# Inferior vena caval filters: 5 years of experience in a tertiary care center

**DOI:** 10.4103/0256-4947.57166

**Published:** 2009

**Authors:** Jalal Saour, Abdulaziz Al Harthi, Mona El Sherif, Ebtisam Bakhsh, Layla Mammo

**Affiliations:** a From the Department of Medicine, King Faisal Specialist Hospital and Research Centre, Riyadh, Saudi Arabia; b From the Department of Medicine, King Fahad Medical City, Riyadh, Saudi Arabia; c From the Department of Biomedical Sciences, University of Detroit Mercy Dental School, Michigan, USA

## Abstract

**BACKGROUND AND OBJECTIVES:**

Interruption of the Inferior Vena Cava (IVC) is recommended in certain cases to prevent Pulmonary Embolism (PE). Reported data on the efficacy and rate of complications vary considerably.

**PATIENTS AND METHODS:**

We conducted a retrospective analysis of patients who had a temporary or permanent IVC filter inserted at our institution during the past 5 years.

**RESULTS:**

Seventy-seven of 225 patients (34%) with Venous Thrombosis (VT) had an IVC filter inserted. Deep vein thrombosis and PE were the most common causes for anticoagulation. Bleeding was the reason for IVC filter insertion in 48 (62%). The only complication found was the breaking of a temporary filter during removal related to the procedure. However, 3 patients (out of 10) had a recurrence of VT after prolonged discontinuation of anticoagulation.

**CONCLUSIONS:**

Our criteria for indication of IVC filter insertion are in line with current standard of care. The immediate and delayed complications caused by IVC filter insertion was low. Active bleeding was the most common indication for filter insertion, whereas inherited thrombophilia was relatively common.

Venous thromboembolism (VTE) is a significant cause of morbidity and mortality. Pulmonary embolism (PE) is the most serious complication of VTE. It affects more than 350 000 patients in the USA annually and results in as many as 240 000 deaths per year.^1^ Despite improvement in diagnosis and management, the average annual incidence of VTE in communities has remained stable.^2^ Pulmonary embolism is usually managed with anticoagulation therapy.^3^ Thrombolysis is used for massive and sometimes for submassive PE. When pharmacological therapy fails, or is contraindicated, or in situations where a small additional PE can cause significant deterioration or even death, then interruption of the inferior vena cava (IVC) is recommended.^4^

Mechanical interruption of blood flow as a mean of preventing PE has a long history. Femoral vein ligation first performed by John Junter in 1874, and later advocated by Homans in 1934, failed to prevent PE.^5^ IVC ligation was later tried, but caused major complications.^6^ Mobin-Ubbin filter, the first intraluminal umberella led to significant thrombosis and migration.^7^ In 1973, the Greenfield filter was inserted percutaneously.^7^ Its success was followed by the introduction of several other IVC filters, but the “ideal device” is yet to be found. The recommendations by the Seventh American College of Chest Physicians Conference on Antithrombotic Therapy for IVC filter use states that 1) for most patients with deep vein thrombosis (DVT), routine use of IVC filter is not indicated and 2) placement of an IVC filter is suggested in patients with a contraindication to or a complication of anticoagulation, as well as in those with recurrent thromboembolism despite adequate anticoagulation.^4^ Other less common indications include chronic recurrent PE with severe pulmonary hypertension in patients undergoing surgical embelectomy/endarterectomy, and in patients with a free-floating ileocaval clot.

Data on the efficacy and complications for each filter suffer many limitations and show extreme variability in different series.^9^ It is therefore important that each center look at their own experience with IVC filter insertion and incorporate that into the decision-making process for using such devices. Our objective was to study our experience with IVC filters by looking at the types of filters inserted, the reasons for insertion, complication rates and the safety and efficacy of IVC filters.

## PATIENTS AND METHODS

We conducted a retrospective analysis of patients who had a temporary or permanent filter inserted at King Faisal Specialist Hospital and Research Centre in the past 5 years (2002-2007). The study was approved by the Institute Research Advisory Council and the Bioethics Committee. A “Data Acquisition Form” was developed by the investigators that allowed the incorporation of all relevant data. The complications recorded included radiological complications, bleeding, hematoma, failure of insertion, infection, migration and thromboembolism.

## RESULTS

Seventy-seven patients were identified, including 42 males (54.5%) and 35 females (45.5%). Sixty-six patients (85.7%) were Saudis and 11 (14.3%) were of mixed nationalities. Forty-five patients (58.4%) were followed for 5 years, seven patients (9%) for four years. Ten were lost to follow-up, five of whom had cancer and who lived outside Riyadh and probably died. The minimal follow-up period for these 10 patients was six months during which no complications related to the IVC filter were reported. Sixteen patients died, with sepsis, multiorgan failure or cancer within two weeks of the IVC filter insertion. The majority (14/16) died while in the intensive care unit.

Of the 77 IVC filters inserted, 50 were permanent filters and 27 were temporary. Twenty of the 27 temporary filters (74.1%) were inserted in patients with active bleeding (20/77). Seven temporary filters (25.9%) were inserted to protect a high-risk patient from future PE, usually as a perioperative measure. Of the 27 temporary IVC filters inserted, fourteen (51.9%) were later removed.

The reason(s) for anticoagulation is shown in Table 1- some patients had more than one reason for anticoagulation. Table 2 shows the reason for inserting an IVC filter, the most common being major bleeds. Table 3 shows the site of these major bleeds. Table 4 shows the risk factor(s) for venous thrombosis. Most patients had more than one risk factor. The most common risk factor was immobility (41.55%). The reason for immobility was mostly the post-operative period after major surgery (62%), followed by multiple injuries secondary to trauma (37%).

**Table 1:** Reasons for anticoagulation in 77 patients who had IVC filters.

Reason for anticoagulation*	Number of cases
Deep vein thrombosis	27 (35%)
Pulmonary embolism	22 (28%)
Recurrent deep vein thrombosis	5 (6.0%)
Recurrent pulmonary embolism	3 (4%)
Recurrent deep vein thrombosis and pulmonary embolism	8 (10.4%)
Portal vein thrombosis	1 (1.3%)
Inferior vena caval thrombosis	1 (1.3%)
Severe pulmonary hypertension	1 (1.3%)
Deep vein thrombosis prophylaxis	5 (6.0%)
Others	7 (9%)

* Some patients had more than one reason for anticoagulation

**Table 2:** Reasons for IVC filter insertion.

Reason for insertion of IVC filter	Number of cases (%)
Bleeding	48 (62%)
Pre-operative insertion in patients with high risk of venous thrombosis	12 (15.6%)
Pre-thrombectomy	2 (2.6%)
Severe pulmonary hypertension	2 (2.6%)
Severe thrombocytopenia	1 (1.3%)
Recurrent deep vein thrombosis (despite anticoagulation)	4 (5.1%)
Post-operative deep vein thrombosis (high risk of bleeding)	4 (5.1%)
Post-operative pulmonary embolism (high risk of bleeding)	3 (3.9%)
Heparin-induced thrombocytopenia	1 (1.3%)

**Total**	**77**

**Table 3:** Sites of bleeding in patients with IVC filters.

Site of bleeding	Number of cases (%)
Gastrointestinal tract	17 (35.4%)
Intracerebral	9 (18.7%)
Subdural	2 (4.1%)
Subarachoid	1 (2%)
Hematuria	4 (8.3%)
Intraabdominal	1 (2%)
Reteroperitoneal	4 (8.3%)
Pulmonary	1 (2%)
Lower limb	1 (2%)
Tracheostomy	1 (2%)
Vaginal	2 (4.1%)
Multiple Sites	5 (10.4%)

**Total**	**48**

**Table 4:** Risk factors for venous thrombosis in patients with IVC filters.

Risk factor*	Number of cases (%)
Immobility	32
Cancer	26
Post-operative period	20
Trauma	12
Hypercoagulate state	12
Heart failure	5
Critical illness	4
Morbid obesity	4
Stroke	3
Drug abuse	2
Pregnancy	1
HIT	1

* Some patients had more than one risk factor

Seventeen patients out of the 60 (28%) studied (28%) had a hypercoagulable state as shown in Table 5.

**Table 5:** Results of thrombophilia testing (n=60).

Test for thrombophilia	Number of cases
Primary antiphospholid syndrome	5 (8.3%)
Secondary antiphospholipid syndrome	2 (3.3%)
Anti β_2_ glycoprotein	1 (1.7%)
Factor V Leiden (heterozygous)	3 (5%)
Prothrombin 20210G>A mutant gene (heterozygous)	1 (1.7%)
Protein S deficiency	3 (5%)
Antithrombin III deficiency	2 (3.3%)
Protein C deficiency	1 (1.7%)
Essential thrombocytosis	1 (1.7%)

**Total**	**19 (32%)**

*Some patient had more than one risk factor

Anticoagulation therapy was continued after the IVC filter insertion in 22/77 patients (28.5%). All except two had warfarin given to a target INR 2.0-3.0. Two were initially given low molecular weight heparin and later changed to warfarin. Fifty-five (71.5%) patients had their anticoagulation therapy discontinued or not started at all after the IVC insertion because of active bleeding in 48/55 (87%) or a perceived high risk of bleeding if anticoagulants were to be used in 7/55 (12.7%). Of these patients whose anticoagulation therapy was initially discontinued, 45/55 (82%) had their anticoagulation therapy resumed within 10 days as the reason resolved for withholding anticoagulation. Ten patients remained off anticoagulant therapy; 3 developed VT (30%). One was diagnosed with a clot on the IVC, and 2 developed lower limb DVT. In one instance the temporary filter broke during removal and had to be surgically removed. There were no instances of migration, infection or perforation, or death directly related to the procedure.

## DISCUSSION

A recent extensive review of IVC filters showed a widespread variation in complications.^10^ While death due to IVC filter (0.12%) and fatal PE (0.7%) was small, PE post-filter insertion varied from 2% to 5%. Filter fracture (1%) and guide wire entrapment (<1%) were also universally rare. On the other hand, complications from insertion including bleeding, infection, pnuemothorax, stroke, air embolism, filter malposition and malfunction varied considerably, from 4% to 11%. Furthermore the rate of migration of the filter (3% to 69%), penetration of the IVC (9% to 24%), venous access site thrombosis (2% to 28%), obstruction of the IVC (6% to 30%) and venous insufficiency (5% to 59%) showed marked variation.^10^,^11^ Reporting thrombosis of IVC filters has been particularly nonuniform and depends on whether or not all patients (as opposed to only symptomatic patients) were studied, the method of investigating possible thrombosis and the duration of follow up.Reports vary widely from 0% to 28%.^11^

The review shows that the practice of IVC filter insertion at our institution, as far as the indications are concerned, are consistent with the accepted recommendations and standard of care.^4^ The vast majority of patients had a contraindication, complication or failure of anticoagulation therapy (52/77). There was however, some unique features in our study population. The preventive IVC filter insertion pre-operatively in the high-risk patients (including two pre-embelectomy procedures) is testament to the high-risk population encountered in a tertiary (compared to a primary of secondary) care hospital. Furthernore, patients with an IVC Filter insertion had a more serious thromboembolic disease with 50% having PE. They also had more serious comorbid conditions with 16/77 (21%) dying of sepsis and multiorgan failure or advanced cancer and none of thomboembolic complications. The vast majority who died (14/16) were in intensive care unit. Bleeding was the most common cause of IVC filter insertion (63%) as in other series. However, there was a relatively large number of patients with central nervous system bleeds (26% of the total bleeds) which relates to an active neurosurgical program at this center.

Our rate of immediate and delayed complications caused by the IVC filter insertion was reassuringly low and should allow us to be more liberal in using the filters, but only for well-defined and accepted indications. One instance of a break in the tip of the filter was the only complication of the procedure per se. However there was a 30% chance of recurrence of VT when an IVC filter was inserted and anticoagulation therapy was discontinued indefinitely and this confirms the need for long-term anticoagulation post-IVC filter insertion.^12^

The number of patients who tested positive for thrombophilia was astonishingly high and requires further study. It is possible that the protein C and protein S deficiency may be acquired (because of Warfarin therapy or other diseases, rather than genetic). However, even if all protein C and S deficient patients are considered to be acquired, we still have a 20% prevalence. Our data from more than 800 healthy Saudi individuals showed a very low incidence of familial thrombophilia.^13^ To our knowledge this is the first report on IVC filters in Saudi Arabia and the Gulf. Our data represent 186 patient-years of experience.

## References

[1] Bick RL (1999). Hereditary and acquired thrombophilia: Preface. Semin Thromb Hemost.

[2] Heit JA, Silverstein MD, Mohr DN, Petterson TM, Lohse CM, O'Fallon WM (2001). The epidemiology of venous thromboembolism in the community. Thromb Haemost.

[3] Barritt DW, Jordan SC (1960). Anticoagulant drugs in the treatment of pulmonary embolism: A controlled trial. Lancet.

[4] Greenfield LJ (1992). Evolution of venous interruption of pulmonary thromboembolism. Arch Surg.

[5] Ochsner A, Ochsner JL, Saunders HS (1970). Prevention of pulmonary embolism by caval ligation. Ann Surg.

[6] Mobin-Uddin K, Smith PE, Martines LO, Lombardo CR, Jude (1967). A vena caval filter for the prevention of pulmonary embolus. Surg Forum.

[7] Tadavarthy SM, Castaneda-Zuniga W, Salomonowitz E, Lund G, Cragg A, Hunter D (1984). Kimray Greenfield vena cava filter: Percutaneous introduction. Radiology.

[8] Buller HR, Agnelli G, Hull RD, Hyers TM, Prin SM, Raskob GE (2004). Antithrombotic therapy for venous thromboembolic disease Seventh ACCP Conference on Antithrombotic and Thrombolytic therapy. Chest.

[9] Kinney TB (2003). Update on Inferior Vena Cava Filters. J Vasc Interv Radiol.

[10] Failla PJ, Reed KD, Summer WR, Karam GH (2005). Inferior vena cava filters: Key considerations. Am J Med Sci.

[11] Strieff MB (2000). Vena caval filters. Blood.

[12] The PREPIC Study Group (2005). Eight year follow-up of patients with permanent vena caval filters in the prevention of pulmonary embolism. Circulation.

[13] Mammo L, Sheereen A, Saour T, Shoukri M, Saour J (2007). Incidence of five prothrombotic gene polymorphism in healthy Saudi Arabians. J Thromb Haemost.

